# Linked CSF reduction of phosphorylated tau and IL-8 in HIV associated neurocognitive disorder

**DOI:** 10.1038/s41598-019-45418-2

**Published:** 2019-06-19

**Authors:** Tugba Ozturk, Alexander Kollhoff, Albert M. Anderson, J. Christina Howell, David W. Loring, Drenna Waldrop-Valverde, Donald Franklin, Scott Letendre, William R. Tyor, William T. Hu

**Affiliations:** 1 Department of Neurology, Emory University School of Medicine, Atlanta, GA USA; 2 Center for Neurodegenerative Diseases, Emory University School of Medicine, Atlanta, GA USA; 3 Department of Medicine – Division of Infectious Disease, Emory University School of Medicine, Atlanta, GA USA; 4 Center for Neurocognitive Studies, Emory University Hodgson Woodruff School of Nursing, Atlanta, GA USA; 5 HIV Neurobehavioral Research Center, University of California, San Diego, CA USA; 6 Atlanta Veterans Affairs Medical Center, Decatur, GA USA

**Keywords:** Dementia, Central nervous system infections

## Abstract

HIV-associated neurocognitive disorder (HAND) is a common condition in both developed and developing nations, but its cause is largely unknown. Previous research has inconsistently linked Alzheimer’s disease (AD), viral burden, and inflammation to the onset of HAND in HIV-infected individuals. Here we simultaneously measured cerebrospinal fluid (CSF) levels of established amyloid and tau biomarkers for AD, viral copy numbers, and six key cytokines in 41 HIV-infected individuals off combination anti-retroviral therapy (14 with HAND) who underwent detailed clinical and neuropsychological characterization, and compared their CSF patterns with those from young healthy subjects, older healthy subjects with normal cognition, and older people with AD. HAND was associated with the lowest CSF levels of phosphorylated tau (p-Tau_181_) after accounting for age and race. We also found very high CSF levels of the pro-inflammatory interferon gamma-induced protein 10 (IP-10/CXCL10) in HIV regardless of cognition, but elevated CSF interleukin 8 (IL-8/CXCL8) only in HIV-NC but not HAND. Eleven HIV-infected subjects underwent repeat CSF collection six months later and showed strongly correlated longitudinal changes in p-Tau_181_ and IL-8 levels (R = 0.841). These data suggest reduced IL-8 relative to IP-10 and reduced p-Tau_181_ to characterize HAND.

## Introduction

HIV-associated neurocognitive disorders (HAND) can affect up to 50% of people living with HIV^1–3^. With more than 1.1 million Americans currently living with HIV^4^, HAND is a more common cognitive disorder than frontotemporal dementia^5^ or Lewy body dementia^6^. In the era of combination anti-retroviral therapy (cART)^7^, severe HIV-associated dementia is less common in developed nations but milder forms of HAND^8–10^ can still interfere with job performance and compliance with the complex multi-drug combination^11^. At the same time, accurate clinical diagnosis of mild HAND (such as asymptomatic neurocognitive impairment and mild neurocognitive disorder)^12^ remains challenging because of common confounds (e.g., substance abuse). Biofluid or imaging markers have the potential of enhancing early and accurate detection of mild HAND. Such biomarkers will be especially important in drug development if they also reflect known disease mechanisms or provide new pathophysiologic insight.

Whereas post-mortem studies commonly associated viral infection as a key mechanism in HIV-associated dementia^13^, no neuropathologic process has been consistently identified in HAND^14^. Gliosis was the most common finding^15,16^, and inclusions associated with age-related neurodegenerative disorders such as Alzheimer’s disease (AD) were variably identified. Cerebral PET imaging for neuritic plaques also failed to show increased amyloid deposition in HAND^17^. On the other hand, some ante-mortem cerebrospinal fluid (CSF) studies examining established AD biomarkers paradoxically showed changes in HAND similar to AD. Two US studies – but not one Dutch study – revealed reduced levels of beta-amyloid 1–42 (Aβ42) suggestive of amyloid deposition^18–20^, while levels of total tau (t-Tau) and tau phosphorylated at threonine 181 (p-Tau_181_) associated with HAND can be increased, normal or reduced^18,21–23^. Because we^24,25^ and others^26^ have shown that African American race and CSF proteins also influence measured CSF AD biomarker levels, an investigation into the relationship between these biomarkers and HAND must account for race and age by recruiting the appropriate comparison groups. Taking advantage of the standardized biofluid collection, handling, and analysis protocols across the HIV and Cognitive Clinics at Emory University, we analyzed AD biomarkers in a cohort of 143 subjects, including 41 infected with HIV who were off (n = 39) or naïve to cART (n = 2), 33 young healthy subjects with normal cognition (NC_young_), 44 older healthy subjects with normal cognition (NC_older_), and 25 older subjects with mild cognitive impairment or mild dementia due to AD (MCI/AD). We further explored whether CSF AD biomarker levels in HAND were influenced by viral load and altered levels of key CSF cytokines linked to innate immunity or T-cell-related pathways (selected for biological function and reproducibility in CSF assays), including interferon-γ-inducible protein 10 (CXCL10/IP10), interleukin 8 (CXCL8/IL-8), fractalkine (CX3CL1), tumor necrosis factor alpha (TNF-α), macrophage-derived chemokine (MDC), and interleukin 10 (IL-10). Finally, to lend support for a mechanistic link between AD biomarkers and inflammation, we examined in a group of HIV subjects with serial CSF collection whether the two biomarker sets underwent parallel changes over time.

## Results

### HAND was associated decreased CSF p-Tau_181_ levels

**Figure 1:**
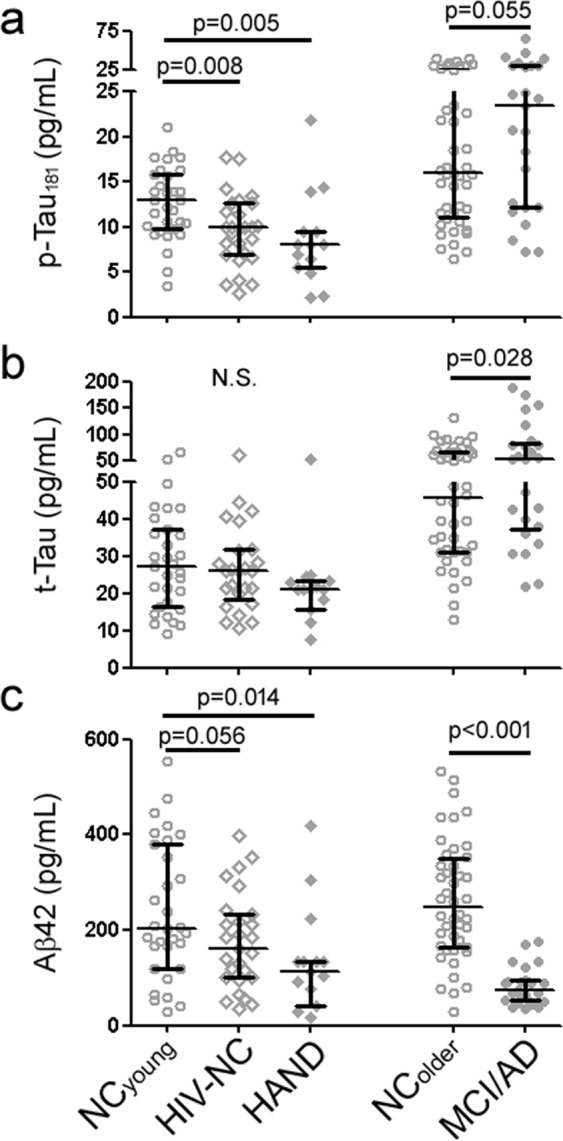
CSF AD biomarkers according to cognition and HIV. HIV-NC and HAND subjects had lower CSF p-Tau_181_ levels than NC_young_ (**a**), but not t-Tau levels (**b**). HAND subjects also had lower CSF Aβ42 levels than NC_young_ (**c**). For comparison, MCI/AD subjects had higher p-Tau_181_ and t-Tau levels, and lower Aβ42 levels than NC_older_. Bars represent median and interquartile range.

We first compared levels of CSF AD biomarkers (Aβ42, t-Tau, p-Tau_181_) according to cognition (normal vs. impaired; see Methods), HIV status (infected or not-infected), and age (Table 1). Consistent with the positive correlation between tau markers and age from a large European cohort of healthy middle-aged adults^27^, NC_older_ had higher t-Tau and p-Tau_181_ levels than NC_young_. Among younger subjects (<60 years of age), HIV with normal cognition (HIV-NC) and HAND were both associated with lower p-Tau_181_ and Aβ42 levels than NC_young_ (Fig. 1a–c, no significant differences in t-Tau levels). Analysis of co-variance (ANCOVA) in the whole cohort (n = 143) showed African American race (F(3, 138) = 14.798, p < 0.001)^25^, having HIV (F = 7.249, p = 0.008), and having HAND (represented by the interaction term between cognitive impairment and having HIV, F = 4.764, p = 0.031) were independently associated with reduced p-Tau_181_ levels, while older age (F = 7.066, p = 0.009) and cognitive impairment due to AD (F = 2.982, p = 0.086) were associated with increased p-Tau_181_ levels. For Aβ42, lower levels were associated with cognitive impairment (F = 21.205, p < 0.001), cognitive impairment due to AD (F = 7.922, p = 0.006), and female gender (F = 8.207, p = 0.005). Hence, reduced p-Tau_181_ was a feature of both HIV (regardless of cognition) and additionally HAND, while reduced Aβ42 was associated specifically with AD and non-specifically with cognitive impairment. Importantly, there was a strong correlation between Aβ42 and p-Tau_181_ levels in the younger population, which has not been previously reported in older healthy controls from AD studies. A review of prior studies which identified HIV/HAND-associated alterations in CSF AD biomarkers also showed four out of five previous studies to report changes in Aβ42 and tau along the same direction (three showing paralleled decreases, Table 2, in contrast to the opposite directions of change in AD)^18,19,28,29^. Taken together, we propose that reduced Aβ42 levels in HAND are best interpreted as surrogates for reduced p-Tau_181_.

**Table 1:** Demographics of subjects included in the study (NC_young_: subjects with normal cognition under the age of 60; HIV-NC: HIV-infected subjects with normal cognition; HAND: HIV-infected subjects with cognitive impairment; NC_older_: subjects with normal cognition at or over the age of 60; MCI-AD: subjects with mild cognitive impairment and CSF biomarkers consistent with Alzheimer’s disease). *p ≤ 0.003 vs. NC_young_.

	NC_young_ (n = 31)	HIV-NC (n = 27)	HAND (n = 14)	NC_older_ (n = 44)	MCI/AD (n = 25)
Male (%)	21 (68%)	22 (82%)	11 (79%)	20 (45%)	12 (48%)
African American (%)	13 (42%)	24 (89%)*	12 (86%)*	19 (43%)	12 (48%)
Age (SD), yr	36.9 (11.1)	40.8 (8.7)	38.0 (10.0)	71.1 (6.4)*	72.0 (11.1)*
Education (SD), yr	15.0 (2.4)	13.3 (2.5)	13.0 (2.2)	16.8 (2.4)*	16.7 (2.6)
Age of diagnosis (SD), yr	N.A	31.2 (6.5)	27.3 (15.0)	N.A	N.A
Disease duration (SD), yr	N.A	9.4(8.8)	10.2 (9.4)	N.A	N.A
Time since last cART (SD), mo	N.A.	14.9 (13.5)	12.6 (11.1)	N.A.	N.A.
Log_10_(plasma viral load)	N.D.	4.46 (0.80)	5.12 (0.85)	N.D.	N.D.
Log_10_(CSF viral load)	N.D.	2.97 (1.05)	3.28 (1.03)	N.D.	N.D.
CSF AD biomarkers (SD)					
Aβ42, pg/mL	238 (144)	176 (100)	139 (111)*	262 (124)	83 (39)*
t-Tau, pg/mL	28.6 (13.3)	25.6 (12.0)	22.1 (10.1)	51.4 (25.8)*	71.5 (48.5)*
p-Tau_181_, pg/mL	12.7 (4.0)	9.6 (3.9)	8.7 (5.2)*	18.8 (9.5)*	24.4 (14.2)*
CSF cytokines (SD)					
IL-8, pg/mL	66 (17)	113 (49)*	79 (20)	92 (27)*	79 (23)
IP-10, ng/mL	2.5 (3.1)	11.5 (5.9)*	10.7 (6.3)*	4.2 (2.3)	3.2 (1.4)
Fractalkine, pg/mL	142 (37)	177 (33)*	171 (26)*	73 (12)*	73 (13)*
TNF-α, pg/mL	1.8 (0.9)	4.5 (3.3)*	4.7 (4.5)*	2.2 (0.8)	2.1 (0.9)
MDC, pg/mL	86 (46)	194 (229)*	123 (124)*	175 (78)*	189 (101)*
IL-10, pg/mL	5.2 (2.7)	8.8 (8.5)*	6.8 (4.6)	7.1 (1.6)	6.8 (1.9)

**Table 2:** Previous studies involving CSF AD biomarker or cytokine alterations in HIV-NC, HAND, or HIV-associated dementia (HAD).

Reference	Assay	Analytes	NC (pg/mL)	HIV-NC (pg/mL)	HAND/HAD (pg/mL)	Key Findings
Ellis, 1998^67^ (US)	ELISA (Innotest)	t-Tau	223 ± 106	185 ± 83		No difference between groups
Brew, 2005^22^ (unknown)	ELISA (Innotest)	Aβ42	544	384	153	Lower Aβ42 in HAD than NC (p < 0.0001) or HIV-NC (p = 0.038); Greater p-Tau_181_ in HAD than NC, p = 0.01
		t-Tau	203	288	314	
		p-Tau_181_	61	74	89	
Clifford, 2009^18^ (US, multi-racial)	ELISA (Innotest)	Aβ42	722	683	522	Lower Aβ42 in HAND than HIV-NC (p = 0.0027); lower t-Tau and p-Tau_181_ in HAND than NC (p = 0.020, 0.044); lower p-Tau_181_ in HIV-NC than NC (p = 0.0026)
		t-Tau	242	183	196	
		p-Tau_181_	47	33	40	
Ances, 2012^17^ (US, multi-racial)	ELISA (Innotest)	Aβ42	699 ± 53	615 ± 69	730 ± 116	Greater Aβ42 in HAND than HIV-NC & Control, p = 0.03
Peluso, 2013^28^ (US, Sweden, Australia)	ELISA (In house)	Aβ42	650	900		Greater Aβ42 (p = 0.0005) and p-Tau_181_ (p = 0.016) in HIV than NC; greater p-Tau_181_ in HIV than NC, p = 0.016
		t-Tau	175	200		
		p-Tau_181_	29	35		
Krut, 2013^29^ (Sweden)	ELISA (custom)	Aβ42	700	N/D	486	Lower Aβ42 in HAD than NC, p < 0.05 Lower p-Tau_181_ in HAD than NC, p < 0.001
		t-Tau	240	N/D	404	
		p-Tau_181_	35	N/D	27	
Mothapo^†^, 2015^20^ (Netherlands)	ELISA (Millipore)	Aβ42	1050	1175	1150	No difference between groups
Cysique, 2015^68^ (Australia)	ELISA (Innotest)	Aβ42	N/A	N/A	N/A	No difference between groups
		t-Tau	N/A	N/A	N/A	
		p-Tau_181_	N/A	N/A	N/A	
Krebs, 2016^30^ (Thailand)	ELISA (Life Tech.)	t-Tau	—	N/A	N/A	No difference between HIV-NC and HAND
de Almeida, 2018^19^ (Brazil)	ECL (MSD)	Aβ42	618	448		Lower Aβ42 (p = 0.002) & t-Tau (p < 0.001), in HIV than NC
		t-Tau	634	198		
		p-Tau_181_	132	164		
Kamat*, 2012^69^ (US multi-racial)	Luminex (BioRad)	IL-8	3	47.7		Greater in HIV than NC, p < 0.001
		IP-10	5	12.5		Greater in HIV than NC, p = 0.001
Mothapo^†^, 2015^20^ (Netherlands)	Luminex (Millipore)	IL-8	25	20	40	Greater IL-8 in HAND than HIV-NC
Yuan^ǂ^, 2015^38^ (China)	Luminex (Millipore)	IL-8	—	22.44	100.6	Greater IL-8 in HAND than HIV-NC p = 0.02
		IP-10	—	5834	17260	Greater IP-10 in HAND than HIV-NC p = 0.01
Krebs, 2016^30^ (Thailand)	Multiplex ELISA (custom)	IL-8	N/A	N/A	N/A	No difference between groups
Mehta^§^, 2017^37^ (US multi-racial)	Luminex (Millipore)	IP-10	—	N/A	N/A	Greater in top than bottom tertile mtDNA (associated with HAND), p < 0.001

When studies did not distinguish between HIV-NC and HAND, mean values of HIV subjects are shown. *55/67 on cART; ^†^5/7 HIV-NC and 14/15 HAND subjects on cART; ^ǂ^17/33 HIV-NC and 20/52 HAND subjects on cART; ^§^158/234 of HIV-NC and 72/98 of HAND subjects on cART. ECL: electrochemiluminescence; ELISA: enzyme-linked immunosorbent assay.

### HAND was associated with higher plasma viral load and relative decrease in IL-8

**Figure 2:**
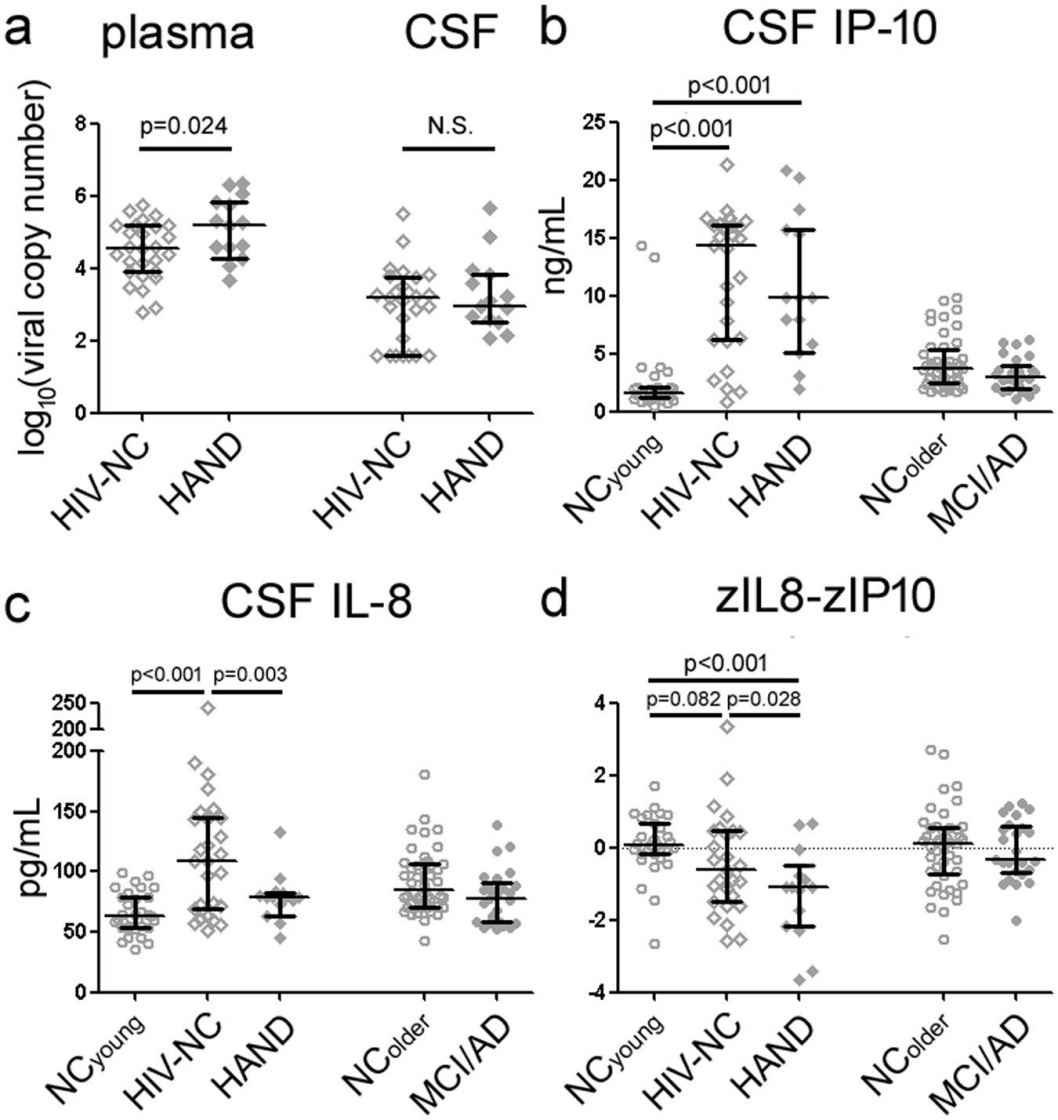
Infectious and inflammatory changes in HIV-NC and HAND. Compared to HIV-NC, HAND was associated with higher plasma viral load and larger proportion of subjects with detectable CSF viral load (**a**). HIV-NC and HAND were both associated with elevated CSF IP-10/CXCL10 (**b**) levels, but CSF IL-8 was only elevated in HIV-NC (**c**). When IP-10 and IL-8 levels were normalized against NC subjects, the difference between IL-8 and IP-10 Z-scores was the most negative in HAND (**d**) while NC_young_, NC_older_, and MCI/AD showed a balance (Z-score difference of 0) between IL-8 and IP-10 (bars represent median and interquartile range).

We next analyzed how HIV infection and neuroinflammation differed according to age, HIV, and cognition. Compared to HIV-NC, HAND subjects had been off cART for similar periods of time prior to neuropsychological and CSF analysis (14.9 vs 12.6 months, p = 0.625). HAND subjects had higher plasma viral load than HIV-NC subjects (log_10_[plasma viral load] of 5.12 vs. 4.46, p = 0.024, Fig. 2a), and a trend of higher CSF viral load (log10[CSF viral load] of 3.17 vs. 2.66, p = 0.167). Entering plasma (F(2, 31) = 0.481, p = 0.494) or CSF (F(2, 31) = 0.001, p = 0.975) HIV titer into the above analysis did not change the effect of HAND on p-Tau_181_.

CSF HIV-NC and HAND were also both associated with higher levels of multiple correlated cytokines than NC_young_, including CSF IP-10 (Fig. 2b, F(2, 138) = 105.875, p < 0.001), fractalkine (F = 54.637, p < 0.001), TNF-α (F = 33.935, p < 0.001), MDC (F = 7.697, p = 0.006), and IL-10 (F = 5.951, p = 0.016). In contrast, IL-8 levels were only elevated in HIV-NC but not HAND (Fig. 2c), with IL-8 levels indistinguishable between NC_young_ and HAND. ANCOVA accounting for age and CSF HIV load among HIV-infected subjects only showed HAND (F(2, 37) = 5.038, p = 0.031) associated with decreased IL-8 levels (CSF – but not plasma – HIV load also associated with greater IL-8 levels, F(2,37) = 3.461, p = 0.071). Therefore, the selectively attenuated or muted IL-8 changes in HIV were a unique cytokine feature for HAND.

Because group-level differences in IP-10 and IL-8 may reflect differential assay performance and other physiologic processes rather than any mechanistic importance related to HAND, we further tested this dissociation between IP-10 and IL-8 according to cognitive impairment in HIV at the individual level. This is especially relevant to resolve conflicting findings on absolute cytokine levels from different studies (Table 2)^20,30^. To do so, we first converted each individual’s IL-8 and IP-10 levels into Z-scores (using NC_young_ and NC_older_ as the normative) to allow for easier comparison of two cytokines’ effect sizes according to each standard deviation alteration^31,32^. We then examined, within each individual, IL-8 and IP-10 levels relative to each other by difference between the two Z-scores (IL-8 and IP-10)^33^. In other word, an individual with proportional increase in IL-8 and IP-10 would have a Z-score difference of 0, while another individual with disproportional increase in IP-10 would have a negative Z-score difference (bias towards IP-10). Through this analysis, we found that HAND was associated with the greatest unopposed change in IP-10 relative to IL-8, while the other groups had relatively balanced changes in IL-8 and IP-10 (Fig. 2d).

### Longitudinal p-Tau_181_ and IL-8 level alterations were highly correlated

**Figure 3:**
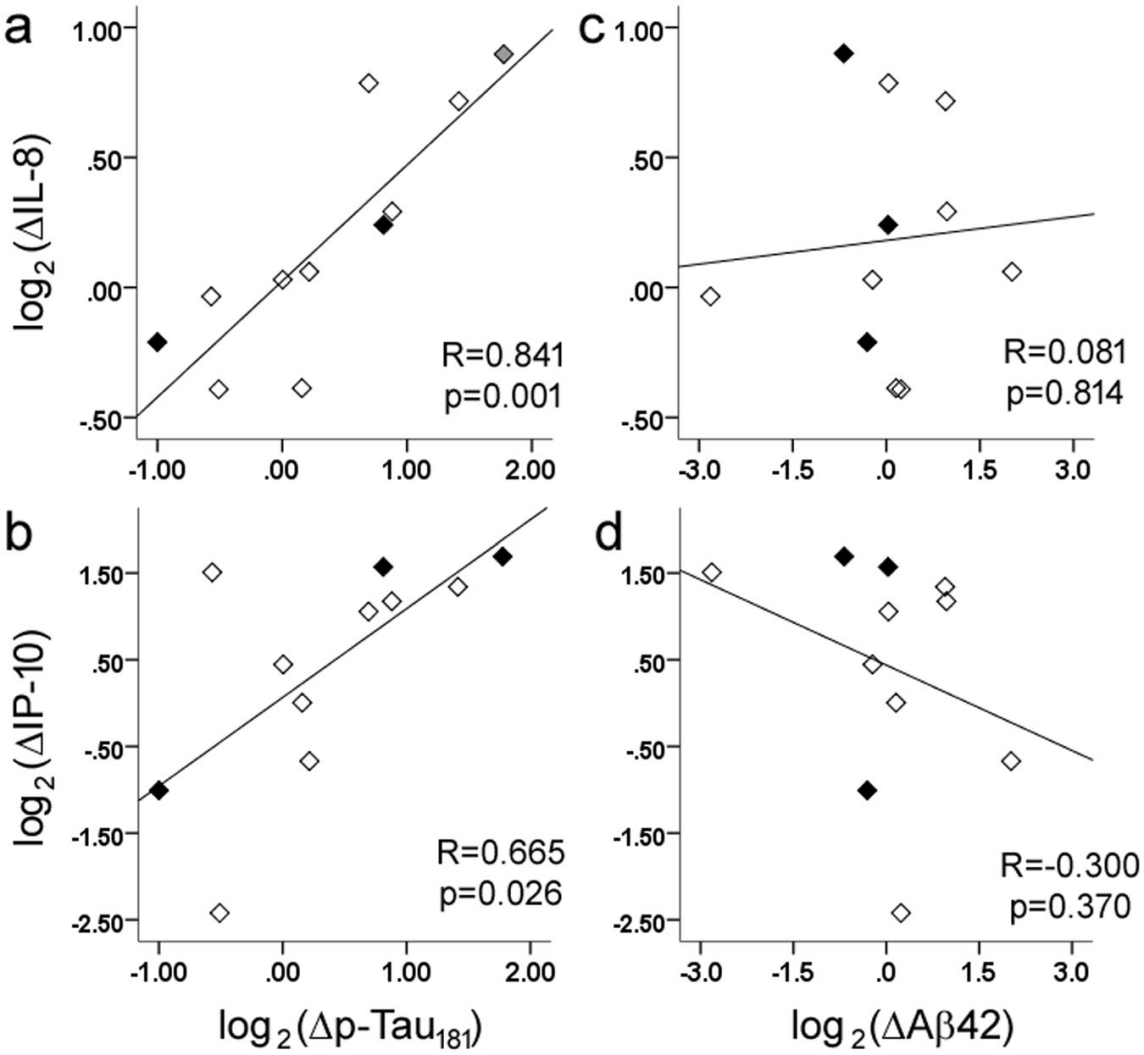
Longitudinal changes in p-Tau_181_ and IL-8 were highly correlated to each other (**a**) in HIV-NC (open diamonds) and HAND (filled diamonds), and to a lesser degree between p-Tau_181_ and IP-10 (**b**, primarily due to association between IP-10 and IL-8). Longitudinal changes in Aβ42 were not correlated to IL-8 (**c**) or IP-10 (**d**). Fold-changes of these markers (∆marker) from baseline CSF sample to follow-up CSF sample were log_2_-tranformed to provide equal weight to increases and decreases. One subject (gray diamond) with HAND at baseline converted to HIV-NC during follow-up.

As HAND was characterized by both reduced p-Tau_181_ levels and a relative reduction in IL-8 (compared to HIV-NC), we next tested whether these two markers undergo paralleled changes over time. Follow-up CSF samples were obtained from eleven subjects (27% of original cohort, including 8 HIV-NC and 3 HAND) six months after the baseline collection. When change in biomarker levels were log_2_-transformed to give equal weight to increases and decreases, we found longitudinal changes in p-Tau_181_ levels to strongly correlate with changes in IL-8 (R = 0.841, p = 0.001, Fig. 3a) and, to a lesser degree, with IP-10 (Fig. 3b), plasma/CSF viral load, or Aβ42. In contrast, longitudinal changes in Aβ42 levels did not correlate with changes in IL-8 (Fig. 3c) or IP-10 (Fig. 3d). Regression analysis showed changes in IL-8, rather than IP-10, best predicted changes in p-Tau_181_. Interestingly, one HIV-infected subject classified as HAND at baseline experienced normalization of his cognitive status during follow-up, and this was accompanied by increases in both his CSF p-Tau_181_ and IL-8 levels (Fig. 3a).

## Discussion

Here we analyzed CSF AD biomarkers and cytokines in a group of HIV-infected and HIV-negative subjects to simultaneously examine two pathways previously implicated in HAND. We identified HAND to be characterized by reduced (but correlated) levels of two AD biomarkers (p-Tau_181_, Aβ42), as well as increased IP-10 levels without accompanying increase in IL-8. These cross-sectional differences may point to a mechanistic link between AD biomarkers and inflammation – at least in HIV, because we also found highly correlated changes in IL-8 and p-Tau_181_ levels over time. Because CSF Aβ42 levels correlated directly with p-Tau_181_ levels in younger subjects regardless of HIV status (rather than inverse pattern in AD), we add to the growing literature that CSF biomarkers do not suggest a link between HAND and AD.

CSF IL-8^34–36^ and IP-10^36,37^ have been independently linked to HIV in the past. To the best of our knowledge, this is the first report of a dissociation between IP-10 and IL-8 changes in HAND. While prior studies consistently showed elevated CSF IP-10 in HIV-NC and HAND^20,37,38^, findings in CSF IL-8 were less consistent with HAND associated with elevated IL-8 in patients from Netherlands^20^ and China^38^ but normal levels in patients from the US^37^. Causes for increased IP-10 and IL-8 levels in HIV are likely complex. There can be migration of peripheral immune cells into the central nervous system (CNS), although the magnitude of change from this is likely limited^39^. Non-HIV infected astrocytes release IL-8 *in vitro* in response to HIV-infected monocyte-derived macrophages^40^, tat protein^41^, and gp120^42^. IL-8 increase in HIV may then reflect the number of non-infected astrocytes capable of responding to the virus. On the other hand, astrocytes genetically engineered to express HIV-1 Nef *in vitro* show markedly elevated IP-10 mRNA and protein levels, in keeping with the post-mortem finding of elevated astrocytic IP-10 in HIV-infected brains^43^. Therefore, the balance between CSF IL-8 and IP-10 levels potentially reflects the health of astrocytes in HIV-infected subjects. Because our study of HIV-infected patients off cART differs in design from prior studies involving mostly of patients on active cART, diminished CSF IL-8 levels in HAND may be reversible with treatment even though this was not universally observed in our small cohort of longitudinally characterized patients. Alternatively, discrepant CSF IL-8 findings in HAND may result from dual infection (more common HAND)^44^ or race-associated differences in *IL8* polymorphism^45,46^. A larger longitudinal CSF study or a quantitative post-mortem analysis of HIV-infected vs. non-infected astrocytes will be necessary to fully test this hypothesis.

CSF AD proteins and cytokines have each been analyzed in HIV and HAND in previous studies (Table 2), but often separately and rarely with longitudinal CSF collection and analysis. Observations linking HAND to AD include *in vitro* evidence of HIV Tat protein^47^ or peptide^48^ inhibiting the primary Aβ42-degrading enzyme neprilysin, and HIV Gag protein mediating the release of Aβ fragments from the amyloid precursor protein^49^. We have ourselves found *in vitro* evidence of mixed neprilysin inhibition by HIV Tat according to enzyme concentration, and acknowledge the potential *in vitro* overlap between HIV proteins and cellular processes capable of clearing and generating AD-related Aβ peptides (unpublished data). However, most studies now (including ours)^18,19,29^ have shown decreases in Aβ42 *and* tau markers, a pattern quite distinct from the AD profile of decreased Aβ42 but increased tau markers. Human studies therefore strongly favor reduced Aβ42 in HAND as a result of younger age instead of AD. While we do not rule out the possibility of cerebral amyloid deposition through non-AD related mechanisms in HAND^17^, the positive identification of reduced p-Tau_181_ levels and decreased IL-8 (relative to IP-10) in HAND warrant further studies on how paired immune dysregulation and tau phosphorylation may contribute to neuronal dysfunction in HIV.

It is not known how higher viral load or reduced IL-8 relative to IP-10 are mechanistically linked to reduced CSF p-Tau_181_ levels. We and others have found aging to be associated with increasing CSF p-Tau_181_ levels, and it is not readily apparent why HAND would be associated with greater youthfulness in the brain. Tau hyperphosphorylation was previously observed in brains of a transgenic rat model of HIV^50^, and a post-mortem study found the greatest brain tau pathology in patients treated with cART. Our observed association between cognitive impairment and low p-Tau_181_ levels in HIV-infected patients off cART is then in keeping with inadequate viral suppression. It is important to note that while elevated t-Tau and p-Tau_181_ levels have been proposed as markers of general neurodegeneration and tau hyperphosphorylation in AD^51^, such relationships are not seen in other common degenerative disorder with rare exception. In fact, we and others have found reduced CSF p-Tau_181_ levels in frontotemporal lobar degeneration with lesions immunoreactive to TDP-43^52,53^, and, to a lesser degree, in Parkinson’s disease^54^. CSF levels of t-Tau and p-Tau_181_ should then not be used as surrogate markers of neurodegeneration in HIV, but HIV does represent a non-AD process that dynamically alters CSF p-Tau_181_ levels at a rate much faster than observed in other conditions. An improved understanding of if and how HIV infection, astrocytic health, and cART modify CSF p-Tau_181_ levels will likely enhance the interpretation of this marker beyond just HIV or HAND.

Among candidate mechanisms capable of modifying tau in HIV, HIV-1 Tat enhances CDK5 translocation^55^ which leads to increased tau phosphorylation, while calcineurin – an abundant brain phosphatase – can dephosphorylate tau. Calcineurin is upregulated *in vitro* by neurons exposed to HIV gp120^56^, and is involved in reactivating latent HIV^57^. Calcineurin inhibition in mice genetically engineered to express gp120 also lead to reduced neuroinflammation and neuropathology^58^. Because of the floor effect in CSF HIV viral load measurements, p-Tau_181_ levels may provide greater sensitivity in monitoring the brain impact of HIV infection especially given the new automated platforms for AD biomarkers^59^. Future studies should incorporate measures of calcineurin-related peptides, markers of neurodegeneration (e.g., neurofilament light chain, neurogranin) as well as p-Tau_181_ and cytokines to better validate this biomarker combination in HIV and HAND.

In sum, we report that HAND is characterized by lower CSF p-Tau_181_ levels, a relative decrease in IL-8 compared to IP-10, and a modest increase in plasma viral load. The strong correlation in the first two markers’ longitudinal changes suggest a shared mechanism independent from plasma viral load, and the identification of fluid biomarkers may help clarify the transition between HIV-NC and HAND. These markers can potentially be used in conjunction with routine viral measures to track insidious cognitive decline, and CSF p-Tau_181_ levels should be tested as a surrogate biomarker of response in clinical trials targeting inflammation in HAND.

## Methods

### Standard protocol approvals, registrations, and patient consents

The studies were approved by the Emory University Institutional Review Board and written informed consent was obtained from all subjects. All experiments were performed in accordance with the US Common Rule (45 CFR Part 46), the Declaration of Helsinki, and Emory University policies.

### Subjects and diagnostic formulation

HIV-infected adult subjects without subacute cognitive decline over the prior 30 days AND were off cART (due to compliance, side effects, or insurance related issues) were recruited at the Emory University Center for AIDS Research (CFAR) as previously described^60^. Briefly, each subject underwent standard medical evaluation and blood tests (TSH, vitamin B12, serum cryptococcal antigen) to rule out reversible causes of cognitive impairment, followed by standardized neuropsychological testing according to the most recent HAND criteria^12^. For this study, participants were excluded if there was a (1) history of neurologic disease known to affect memory (including stroke, malignancy involving the central nervous system, traumatic brain injury, and AIDS-related opportunistic infection in the brain), (2) ongoing substance use including heavy alcohol consumption in the past 30 days (>7 drinks per week for women and >14 drinks per week for men), (3) serious mental illness (e.g, schizophrenia and bipolar disorder; depression was not an exclusion criterion), or 4) history of treated syphilis and a persistently positive rapid plasma regain titer of 1:4 or less. HIV-infected subjects were further invited to return for a 6-month follow-up visit, and 11 individuals returned for the follow-up visit and underwent a second CSF collection.

All HIV-infected participants underwent comprehensive neuropsychological analysis for the assessment of HAND using 14 tests commonly used in studies of cognition and HIV^61^: (1) Trailmaking Part A, (2) Trailmaking Part B, (3) Hopkins Verbal Learning Test total learning, (4) Hopkins Verbal Learning Test delayed recall, (5) Brief Visuospatial Memory Test-Revised learning, (6) Brief Visuospatial Memory Test-Revise delayed recall, (7) Grooved Pegboard (dominant), (8) Grooved Pegboard (non-dominant), (9) Finger Tapping; (10) Stroop Color Naming, (11) Stroop Color-Word interference, (12) Symbol Digit Modalities Test, (13) Letter Fluency (Controlled Oral Word Association Test), and (14) Category fluency (animals). These tests were selected in order to examine at least five domains as recommended. Age, sex-, race-, and education- adjusted T-scores across 14 subtests were averaged to generate a composite neuropsychological score (NPT-14), and the validated global deficit score (GDS) was also calculated with a score ≥ 0.5 to indicate cognitive impairment^62^. A modified standard regression-based correction was used to account for longitudinal practice effect when determining changes in neuropsychological performance in follow-up^63^.

NC_young_ were also recruited at CFAR (n = 13) or as part of a broader biobanking protocol (n = 18) at Emory University. For comparative purposes, we additionally analyzed samples from older African Americans and Caucasians with normal cognition (NC_older_) or MCI/AD (CSF t-Tau/Aβ42 ≥ 0.39 based on a biomarker-autopsy cohort)^64^ recruited under a separate study^25^. All these subjects underwent standardized clinical examination, neuropsychological analysis, consensus diagnosis process, and MR imaging as previously described^25,65^.

### Procedures

In all subjects, plasma and CSF samples were obtained on the same day as neuropsychological analysis^60^. Plasma samples were centrifuged, aliquoted, labeled, and stored at −80 °C until analysis. CSF was collected using a modified Alzheimer’s Disease Neuroimaging Initiative protocol with minor modifications. At CFAR (all HIV subjects and 17 NC_young_), CSF was obtained without overnight fasting using a 20-Gauge cutting edge needle directly into a 15 mL polypropylene tube, centrifuged to remove cellular debris (1800 RPM for 10 min), aliquoted, labeled, and immediately frozen. For other subjects, CSF was collected without overnight fasting using a 24-Gauge atraumatic needle directly into a 15 mL polypropylene tube, aliquoted, labeled, and frozen. We previously showed that CSF centrifugation to remove cellular debris did not influence measured cytokine levels, and samples collected with and without centrifugation can be analyzed together^32,66^.

### Plasma and CSF viral load

Plasma and CSF HIV RNA levels were measured in the real-time HIV-1 RT-PCR assay (Abbott Molecular, Chicago, IL), with the lowest limit of HIV detection at 40 copies/mL. Plasma and CSF viral load were log_10_-transformed because of their non-normality.

### CSF biomarkers

CSF AD biomarker (Aβ42, t-Tau, p-Tau_181_) levels were measured in a Luminex 200 platform using Alzbio3 (Fujirebio USA, Malvern, PA) as previously described^25,52^. Our center achieves an average inter-plate coefficient of variation (CV) of 13% for Aβ42, 10% for t-Tau, and 11% for p-Tau_181_. Levels of six CSF cytokines (IP10, IL-8, fractalkine, TNF-α, MDC, IL-10) were measured using Milliplex MAP Human Cytokine/Chemokine Magnetic Bead Panel (Millipore Sigma, Burlington, MA) in a Luminex 200 platform following the manufacturer’s protocol except two 100 μL aliquots of CSF were used for duplicates. These cytokines were previously selected from a larger panel because of assay sensitivity and intermediate precision in CSF cytokine analysis. In our laboratory, we achieve average intermediate precision (in experiments performed over 9 days) of 9.4% for TNF-α, 12.9% for MDC, 14.7% for IL-7, 4.8% for IP-10, 12.0% for IL-10, 9.2% for IL-9, and 7.6% for IL-8. Freeze-thawing experiment using CSF from six separate subjects showed significant degradation over two freeze-thaw cycles for MDC and TNF-α (p = 0.021 and p = 0.012 for slope in exponential decay), and we previously showed light centrifugation to have minimal impact on these CSF cytokine levels.

### Statistical analysis

All statistical analysis was performed using IBM SPSS 24 (Chicago, IL). ANCOVA was used to determine main effects of HIV, cognition, and the interaction term between HIV and cognition on CSF biomarker levels, including p-Tau_181_, t-Tau, Aβ42, IP-10, IL-8, fractalkine, TNF-α, and IL-10. Student’s T-tests were used to analyze differences in log_10_-transformed plasma and CSF viral load between HIV-NC and HAND, and Chi-squared test was used to analyze proportion of HIV-NC and HAND subjects with undetectable levels of CSF viral load.

To calculate Z-scores for IL-8 and IP-10, mean and standard deviation values from a combined cohort of NC_young_ and NC_older_ were calculated for each cytokine, and every individual’s IL-8 and IP-10 levels were transformed into Z-scores^32^. Z-score differences between IL-8 and IP-10 were calculated by subtracting the former’s Z-score by the latter’s Z-score^33^.

Finally, for HIV-infected patients who underwent serial CSF collections, each individual’s biomarker levels from the follow-up visits were divided by his or her baseline CSF levels to determine fold-change in IL-8, IP10, p-Tau_181_, and Aβ42 levels. The fold-change values then underwent log_2_-transformation to give equal weight to increases and decreases. Because viral load was already analyzed after log_10_ transformation, the change in plasma viral load was represented by the difference in serial log-transformed values. Pearson’s correlation analysis was used to determine relationship between longitudinal changes in these biomarkers.

## Data Availability

The de-identified datasets generated and analyzed during the current study are available from the co-corresponding authors on reasonable request.
